# Noise-induced synchrony of two-neuron motifs with asymmetric noise and uneven coupling

**DOI:** 10.3389/fncom.2024.1347748

**Published:** 2024-02-23

**Authors:** Gurpreet Jagdev, Na Yu

**Affiliations:** ^1^Department of Mathematics, Toronto Metropolitan University, Toronto, ON, Canada; ^2^Institute of Biomedical Engineering, Science and Technology (iBEST), Unity Health Toronto, and Toronto Metropolitan University, Toronto, ON, Canada

**Keywords:** network motifs, coupled oscillators, synchrony, asymmetric noise, heterogeneity

## Abstract

Synchronous dynamics play a pivotal role in various cognitive processes. Previous studies extensively investigate noise-induced synchrony in coupled neural oscillators, with a focus on scenarios featuring uniform noise and equal coupling strengths between neurons. However, real-world or experimental settings frequently exhibit heterogeneity, including deviations from uniformity in coupling and noise patterns. This study investigates noise-induced synchrony in a pair of coupled excitable neurons operating in a heterogeneous environment, where both noise intensity and coupling strength can vary independently. Each neuron is an excitable oscillator, represented by the normal form of Hopf bifurcation (HB). In the absence of stimulus, these neurons remain quiescent but can be triggered by perturbations, such as noise. Typically, noise and coupling exert opposing influences on neural dynamics, with noise diminishing coherence and coupling promoting synchrony. Our results illustrate the ability of asymmetric noise to induce synchronization in such coupled neural oscillators, with synchronization becoming increasingly pronounced as the system approaches the excitation threshold (i.e., HB). Additionally, we find that uneven coupling strengths and noise asymmetries are factors that can promote in-phase synchrony. Notably, we identify an optimal synchronization state when the absolute difference in coupling strengths is maximized, regardless of the specific coupling strengths chosen. Furthermore, we establish a robust relationship between coupling asymmetry and the noise intensity required to maximize synchronization. Specifically, when one oscillator (receiver neuron) receives a strong input from the other oscillator (source neuron) and the source neuron receives significantly weaker or no input from the receiver neuron, synchrony is maximized when the noise applied to the receiver neuron is much weaker than that applied to the source neuron. These findings reveal the significant connection between uneven coupling and asymmetric noise in coupled neuronal oscillators, shedding light on the enhanced propensity for in-phase synchronization in two-neuron motifs with one-way connections compared to those with two-way connections. This research contributes to a deeper understanding of the functional roles of network motifs that may serve within neuronal dynamics.

## 1 Introduction

The synchronization of neural oscillations is acknowledged for its role in facilitating communication between neurons (Varela et al., 2001; Fell and Axmacher, 2011), and is essential for higher order cognitive processes including memory formation, motor coordination, and sensory information processing (Varela et al., 2001; Womelsdorf and Fries, 2007; Kawasaki et al., 2018). Noise, prevalent in real neural networks, plays a crucial role in giving rise to emergent dynamics that may serve important physiological functions (Varela et al., 2001; Fell and Axmacher, 2011). One intriguing constructive effect of noise is noise-induced synchrony, wherein noise alone can induce coordinated and synchronized dynamics. This phenomenon has been widely explored, especially in neural networks, as shown by studies such as (Perc and Marhl, 2006; Perc, 2007; Touboul et al., 2020). Network motifs are frequently recurring structural patterns in neuronal networks, commonly regarded as the fundamental building blocks of complex networks (Milo et al., 2002; Reigl et al., 2004). Among network motifs, those featuring two neurons are notably more prevalent than other multi-neuron motifs (Reigl et al., 2004; Song et al., 2005), making them central to shaping the collective behavior of the network. Consequently, this study focuses on the noise-induced synchrony of the most over-represented motifs: two-neuron motifs.

In the context of two-neuron motifs or two coupled oscillators, prior research has extensively studied the noise-induced synchrony under homogeneous configurations such as common noise (where each neuron encounters identical noise), symmetric noise where each neuron has independent noise but with equal noise intensity), and couplings of exactly equal strengths between neurons. These investigations are well-documented in reviews and books (Rosenblum et al., 2001; Pikovsky, 2002; Boccaletti et al., 2006). In real-world or experimental scenarios, however, coupling and noise patterns often deviate from uniformity (Song et al., 2005; Morgan and Soltesz, 2008). Our understanding of the broader implications associated with the effects of asymmetric noise (where each neuron receives independent noise sources with different noise intensities) and uneven coupling (e.g., unequal coupling strength) is limited, as only a few studies have explored the influence of heterogeneity in noise and/or coupling on the dynamics of two coupled neuronal oscillators. For example, the interplay between uneven coupling strengths and symmetric noise in two coupled oscillators has been shown to promote synchronization (Blasius, 2005) and enhance the transmission of sub-threshold external signals (Masoliver and Masoller, 2018). In a pair of coupled oscillators with uneven coupling and asymmetric additive noise (Amro et al., 2015) finds that the phase coherence of one oscillator is a non-monotonic function of the additive noise applied to the other oscillator: as the phase coherence of one oscillator decreases, the other increases.

To address these less explored aspects, this paper investigates the synchronous dynamics of a pair of excitable neurons in the presence of two sources of heterogeneity: asymmetric noise and uneven coupling. Here the excitable neurons are modeled by the normal form of a Hopf bifurcation (HB), a deterministic framework underpinning critical transitions between quiescent and oscillatory states in complex systems. Therefore our findings provide a versatile framework for illustrating a diverse array of dynamical patterns near a HB within a heterogeneous configuration. Given that the two-neuron motif constitutes a fundamental element in a neuronal network, this study contributes valuable insights into the generation of diverse network behaviors in the context of heterogeneity in both noise and coupling. The structure of the remainder of this paper is as follows: Section 2 provides an introduction to the mathematical model and the methodologies used. In Section 3.1, we present the bifurcation diagram of two deterministic oscillators and Section 3.2 introduces noise-induced oscillations. Section 3.3 focuses on demonstrating noise-induced synchrony and how asymmetric noise affects this synchrony. In Section 3.4, we study the effects of the bifurcation parameter on synchronization, and in Section 3.5, we examine the dual effects of uneven coupling and asymmetric noise on synchrony. Finally, Section 4 offers a summary and engages in a discussion of the findings.

## 2 Materials and methods

Synchrony in neural oscillators is not solely determined by their phase; rather, the statistical prevalence of amplitude dynamics influencing synchronization is noteworthy (Gambuzza et al., 2016). This phenomenon, referred to as amplitude-sensitive synchrony, is a distinctive characteristic of oscillators located near an HB. Hence, our research employs the λ−ω system, recognized as a minimal model (or normal form), capable of capturing both amplitude and phase dynamics of an oscillator in the proximity of a HB. Therefore, the insights gained from this study have broader applicability to other dynamical systems situated in the vicinity of a HB.

### 2.1 Model

**Figure 1:**
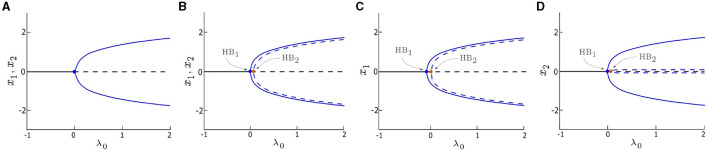
Bifurcation diagrams of the deterministic system (δ_1_ = δ_2_ = 0) vs. control parameter λ_0_. **(A)** single/uncoupled oscillator (*x* = *x*_1_ = *x*_2_) with *d*_1_ = *d*_2_ = 0, a single Hopf bifurcation (HB) occurs at λ = 0. **(B)** symmetrically coupled oscillators (*x*_1_, *x*_2_) with *d*_1_ = *d*_2_ = 0.05, **(C, D)** asymmetrically coupled oscillators (*x*_1_ and *x*_2_, respectively) with *d*_1_ = 0.1 and *d*_2_ = 0.01. Two HB points, HB_1_ and HB_2_, occur and are indicated by blue and orange dots, respectively. The value of λ_0_ corresponding to HB_1_ is zero in **(B–D)**. Stable solutions are marked by solid blue lines and solid black lines, and unstable solutions are marked by dashed blue lines and dashed black lines. Other parameters are: α = −0.2; γ = −0.2; ω_0_ = 2; and ω_1_ = 0.

We examine a duo of coupled λ−ω oscillators, selected with specific parameters that position the model near a supercritical HB. In the absence of noise, these oscillators remain quiescent, but they become excited upon the introduction of an intrinsic noise stimulus. Their coupling strength is uneven, and they are subjected to asymmetric intensities of additive noise. Moreover, both oscillators are represented by the set of stochastic differential equations (SDEs).


(1) $$\begin{array}{l} dx_{i}=\left[\lambda \left(r_{i}\right)x_{i}-\omega \left(r_{i}\right)y_{i}+d_{i}\left(x_{j}-x_{i}\right)\right]dt+\delta_{i}d\eta_{i}\left(t\right) \end{array}$$

(2) $$\begin{array}{l} dy_{i}=\left[\omega \left(r_{i}\right)x_{i}+\lambda \left(r_{i}\right)y_{i}+d_{i}\left(y_{j}-y_{i}\right)\right]dt \end{array}$$

(3) $$\begin{array}{l} r_{i}^{2}=x_{i}^{2}+y_{i}^{2} \end{array}$$

where *i, j* = 1, 2. $r_{i}=\sqrt{x_{i}^{2}+y_{i}^{2}}$ represents the amplitude of the *i*th oscillator. $\lambda \left(r_{i}\right)=\lambda_{0}+\alpha r_{i}^{2}+\gamma r_{i}^{4}$ controls the increment and decrement of the amplitude of the *i*th oscillator. λ_0_ is the control parameter and a HB occurs at λ_0_ = 0 (see Figure 1A). $\omega \left(r_{i}\right)=\omega_{0}+\omega_{1}r_{i}^{2}$ determines the increment and decrement of the frequency of the *i*th oscillator. We consider a supercritical HB by setting the parameter values as α = −0.2, γ = −0.2, ω_0_ = 2, and ω_1_ = 0. *d*_*i*_(*x*_*j*_ − *x*_*i*_) and *d*_*i*_(*y*_*j*_ − *y*_*i*_) represent diffusive coupling between oscillators *i* and *j* with oscillator specific coupling strength, *d*_*i*_, *i* = 1, 2. δ_*i*_*dη*_*i*_(*t*) represents an independent intrinsic noise applied to *x*_*i*_ (i.e., the noise is unique to each oscillator) where η_*i*_(*t*) is a Wiener process with zero mean and unity variance and δ_*i*_ is the noise intensity. We restrict our attention to the excitatory coupling in the range 0.01 ≤ *d*_1_, *d*_2_ ≤ 0.3 (as in Yu et al., 2008).

### 2.2 Methods

To study the interplay of noise and coupling and its effect on the synchronization of our model we analyze the synchrony of both oscillators when subject to the additive noise, δ_*i*_*dη*_*i*_, *i* = 1, 2. Since the oscillators rotate about the fixed point (*x*_*i*_, *y*_*i*_) = (0, 0), *i* = 1, 2, when driven by noise, the time-dependent phase of each oscillator is taken to be the natural phase (Rosenblum et al., 2001),


(4) $$\begin{array}{l} \phi_{i}\left(t\right)=\arctan\left(y_{i}\left(t\right)/x_{i}\left(t\right)\right) \end{array}$$

*i* = 1, 2. In the classical treatment of phase analysis, synchrony measures are often based on the distribution of the phase difference, Δϕ_*n,m*_ = *nϕ*_2_ − *mϕ*_1_, where *n, m* ∈ ℕ characterize the order of locking (Rosenblum et al., 2001, 2022). However, in the presence of noise, the phase of the oscillators can exhibit random jumps of ±2π, called phase slips, which can cause the phase difference, Δϕ_*n,m*_, to compound errors, and lead to erroneous results. Therefore, instead of considering the natural phase in Equation 4, we consider the cyclic relative phase (Mormann et al., 2000; Rosenblum et al., 2001),


(5) $$\begin{array}{l} \varphi_{i}\left(t\right)=\phi_{i}\left(t\right)\text{mod}2\pi \end{array}$$

which is the natural phase wrapped over the unit circle. This procedure ensures that errors in Δφ_*n,m*_ caused by phase slips do not compound which leads to more stable numerical results. Furthermore, for simplicity, we consider only 1 − 1 synchronization: Δφ = Δφ_1,1_.

**Figure 2:**
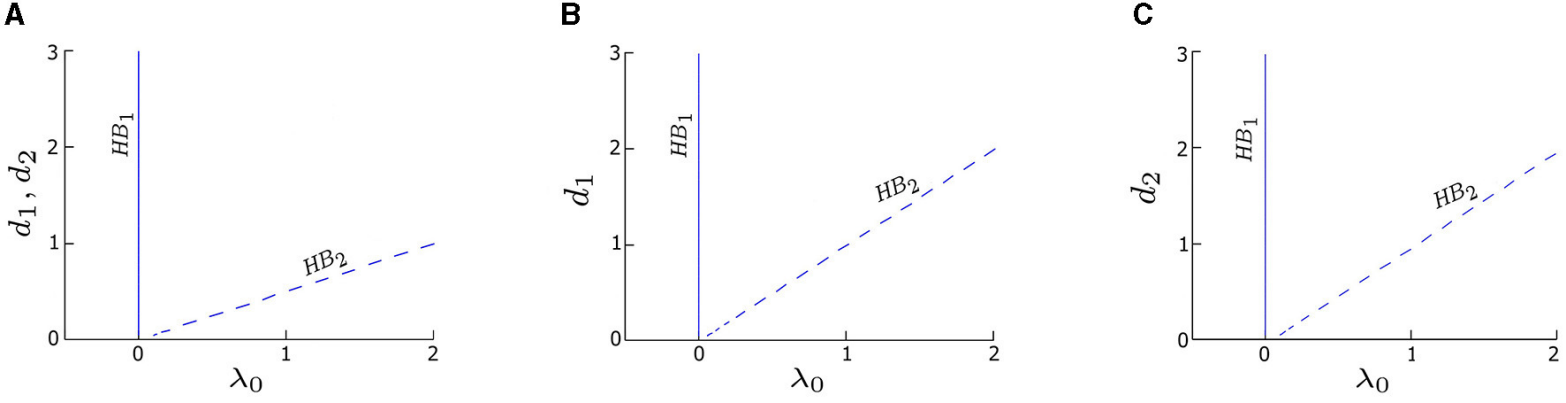
Two-parameter bifurcation diagrams (coupling strength *d*_*i*_ vs. λ_0_) for three coupling cases: **(A)** symmetric coupling strengths, *d*_1_ = *d*_2_, **(B)** asymmetric coupling strengths with varying *d*_1_ and fixed *d*_2_ = 0.05, and **(C)** asymmetric coupling strengths with varying *d*_2_ and fixed *d*_1_ = 0.05. The branches labeled HB_1_ (solid blue) and HB_2_ (dashed blue) represent the same two distinct HB points in Figure 1. The value of λ_0_ corresponding to HB_1_ is consistently zero across three cases, aligning with the observation in Figure 1. Conversely, the value of λ_0_ corresponding to HB_2_ linearly increases with *d*_*i*_. Other parameters are: δ_1_ = δ_2_ = 0; α = −0.2; γ = −0.2; ω_0_ = 2; and ω_1_ = 0.

**Figure 3:**
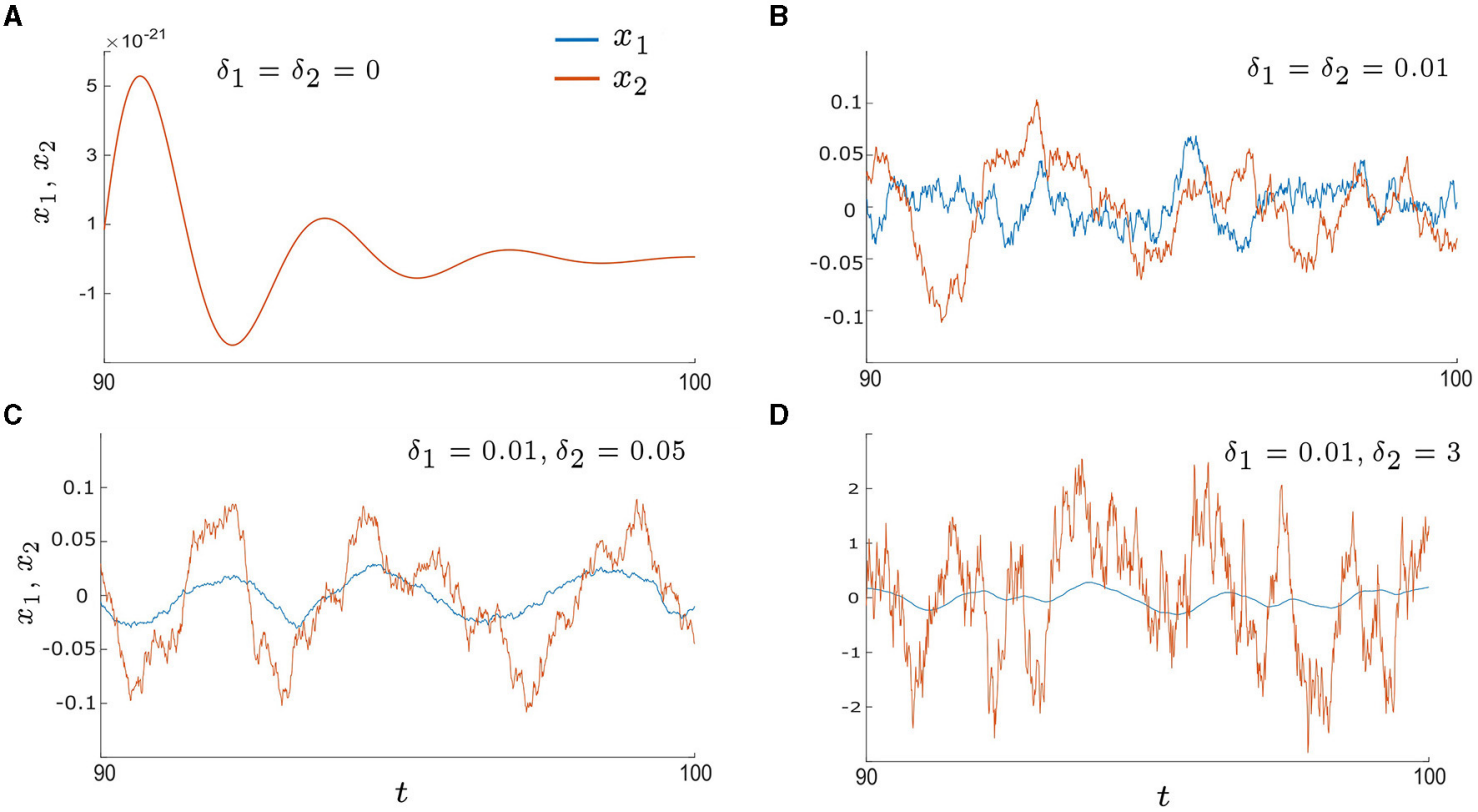
Time series of *x*_1_and*x*_2_ in the excitable regime (λ_0_ < 0) with various noise intensities: **(A)** δ_1_ = δ_2_ = 0, **(B)** δ_1_ = δ_2_ = 0.01, **(C)** δ_1_ = 0.01 and δ_2_ = 0.05, and **(D)** δ_1_ = 0.01 and δ_2_ = 3. The blue lines represent *x*_1_ and the orange lines represent *x*_2_. **(A)** shows the deterministic system with a stable fixed point at *x*_*i*_ = 0, resulting in an overlap between *x*_1_ and *x*_2_. **(B–D)** illustrate the noise-induced oscillations, with **(C)** showing higher levels of regularity and synchrony in comparison to **(B, D)**. Other parameters are: α = −0.2; γ = −0.2; ω_0_ = 2; ω_1_ = 0; λ_0_ = −0.5; *d*_1_ = 0.3; and *d*_2_ = 0.01.

**Figure 4:**
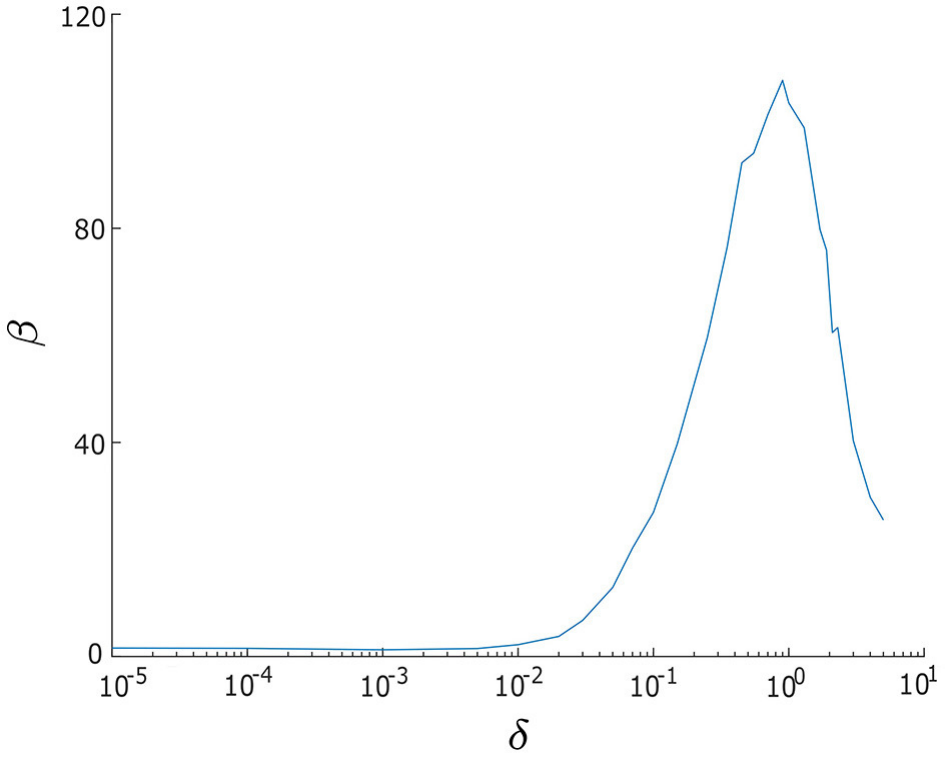
Signal-to-noise ratio measure β vs. noise intensity δ for a single/uncoupled neuron oscillator in the excitable regime (λ_0_ < 0). Greater regularity in noise-induced oscillations is indicated by larger values of β. The largest β occurs when noise intensity is δ = 0.9. Other parameters are: α = −0.2; γ = −0.2; ω_0_ = 2; ω_1_ = 0; and λ_0_ = −0.5.

**Figure 5:**
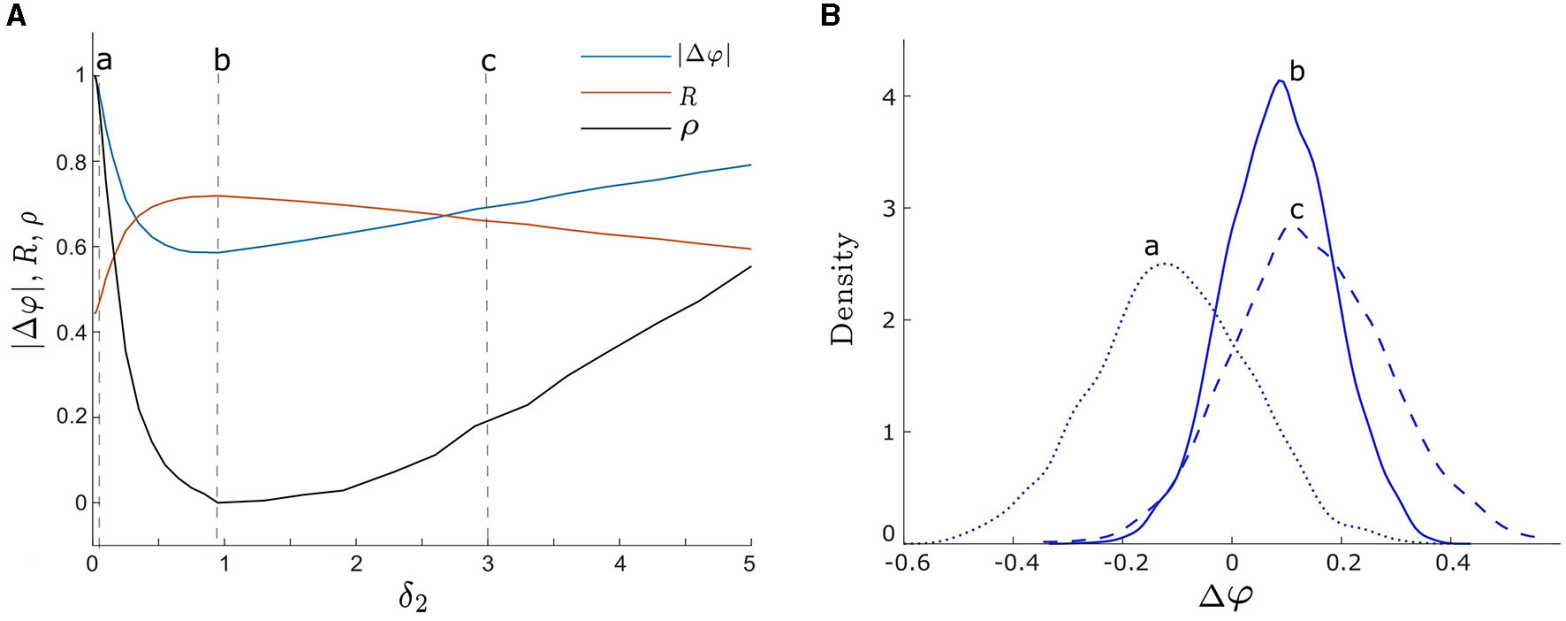
**(A)** Three measures of phase synchronization: |Δφ| (the absolute value of the phase difference), *R* (the mean phase coherence), and ρ (the synchronization index) vs. δ_2_. The orange, blue, and black curves correspond to *R*, |Δφ|, and ρ, respectively. The three vertical dashed lines labeled by a, b, and c correspond to δ_2_ = 0.05, 0.95, and3, respectively. Smaller values of |Δφ| and ρ, and larger values of *R* indicate greater noise-induced synchrony. All three measures demonstrate consistent synchrony dynamics as δ_2_ varies, reaching maximum synchrony at δ_2_ = 0.95. **(B)** The empirical probability density functions of Δφ, with the curves a, b, and c corresponding to noise intensities δ_2_ = 0.05, 0.95, and 3 as in **(A)**, respectively. The peaks of density functions corresponding to a, b, and c are Δφ = −0.12, 0.08, and 0.12, respectively. Curve b with δ_2_ = 0.95 exhibits the most concentrated distribution of Δφ, indicating optimal noise-induced synchrony, consistent with the finding in **(A)**. Other parameters are: δ_1_ = 0.05; α = −0.2; γ = −0.2; ω_0_ = 2; ω_1_ = 0; λ_0_ = −0.5; *d*_2_ = 0.01; and *d*_1_ = 0.3.

**Figure 6:**
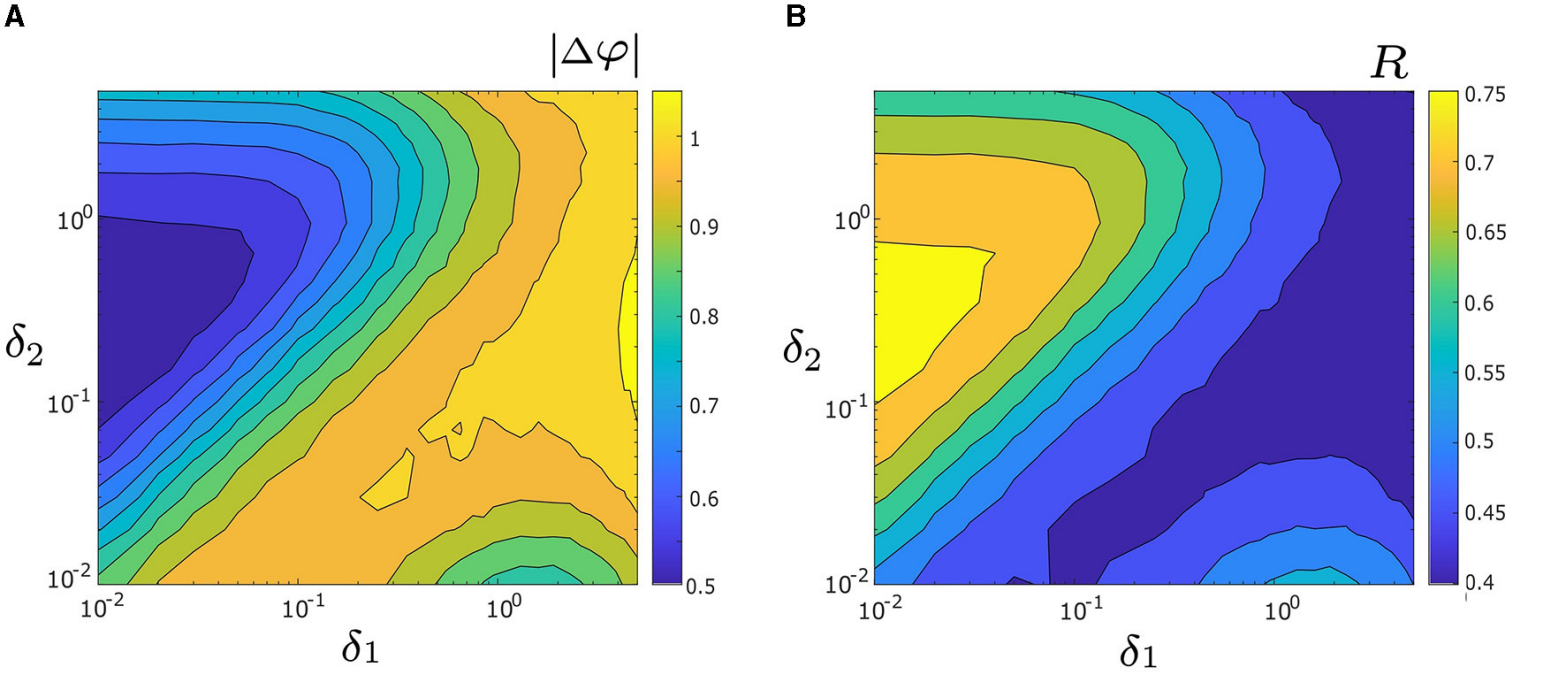
**(A)** Heat map of the mean absolute phase difference |Δφ|, as a function of noise intensities δ_1_ and δ_2_. Colder colors correspond to smaller values of |Δφ| and a decrease in |Δφ| indicates greater synchrony. The maximum synchrony is located within the dark blue triangle on the left side. The three-dimensional visualization of (A) is depicted in Supplementary Figure 1A. **(B)** Heat map of mean phase coherence, *R*, as a function of δ_1_ and δ_2_. Warmer colors correspond to larger values of *R* and an increase in *R* indicates better noise-induced synchrony. The maximum synchrony is within the yellow triangle on the left side, consistent with the observation in **(A)**. Other parameters are: α = −0.2; γ = −0.2; ω_0_ = 2; ω_1_ = 0; λ_0_ = −0.5; *d*_1_ = 0.3; and *d*_2_ = 0.01.

**Figure 7:**
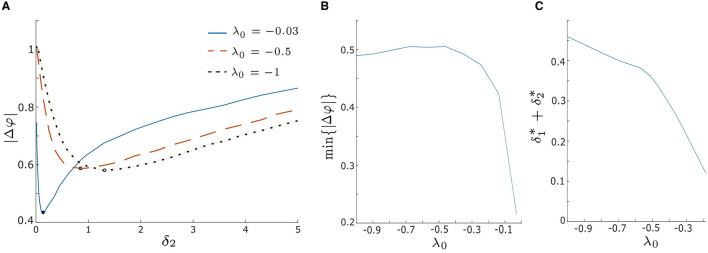
**(A)** Absolute value of the phase difference, |Δφ|, vs. noise intensity δ_2_ for λ_0_ = −0.03, −0.5, and − 1 with δ_1_ = 0.1. The blue solid, orange dashed, and black dotted lines correspond to λ_0_ = −0.03, −0.5, and − 1, respectively. Increasing λ_0_ leads to a decrease in the minimum value of |Δφ|, implying enhanced synchrony as λ_0_ approaches zero. **(B)** Minimum |Δφ|, denoted as min{|Δφ|}, as a function of λ_0_ by varying both δ_1_ and δ_2_ over from 0.01 to 5. min{|Δφ|} significantly decreases when λ_0_ > −0.3, suggesting a rapid improvement in synchrony as λ_0_ approaches zero. **(C)** The sum of the optimal noise intensities, $\delta_{1}^{*}+\delta_{2}^{*}$, decreases as λ_0_ increases, indicating that smaller noise intensities are needed to achieve min{|Δφ|} as λ_0_ approaches zero. Other parameters are: α = −0.2; γ = −0.2; ω_0_ = 2; ω_1_ = 0; *d*_1_ = 0.3; and *d*_2_ = 0.01.

**Figure 8:**
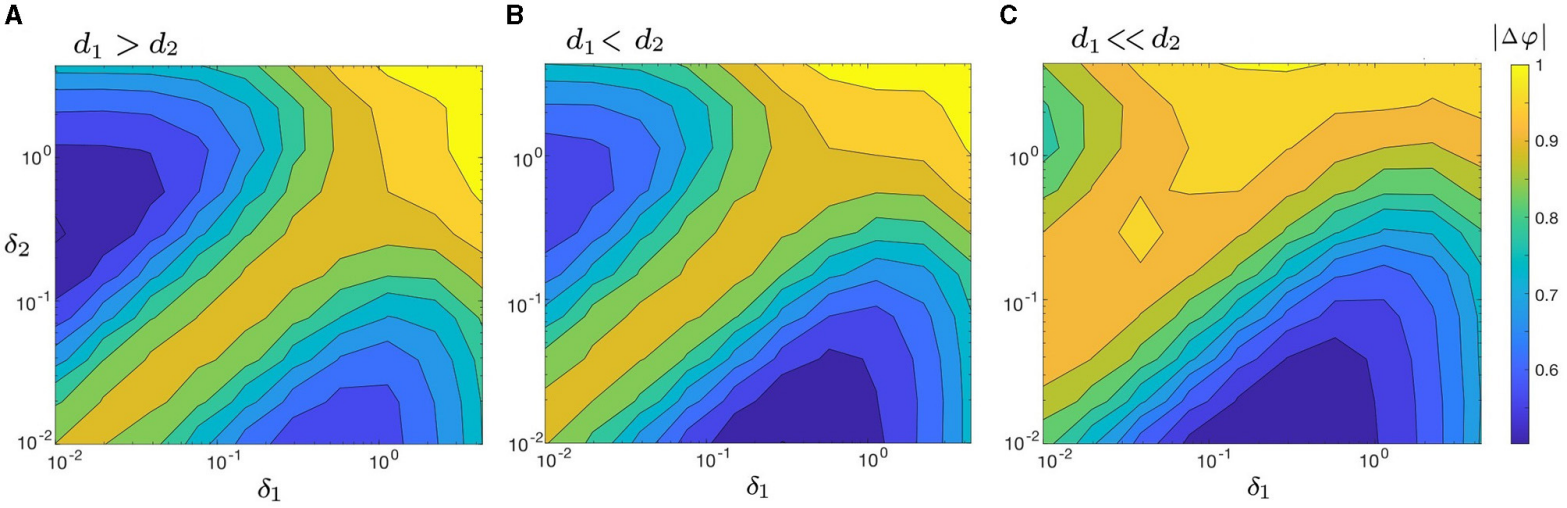
Heat maps of |Δφ|, as a function of δ_1_ and δ_2_ for three cases of coupling strengths: **(A)** *d*_1_ > *d*_2_ (with *d*_1_ = 0.2, *d*_2_ = 0.1); **(B)** *d*_1_ < *d*_2_ (with *d*_1_ = 0.1, *d*_2_ = 0.2); and **(C)** *d*_1_ ≪ *d*_2_ (with *d*_1_ = 0.01, *d*_2_ = 0.3). When *d*_1_ ≫ *d*_2_ (Figure 6A) and *d*_1_ > *d*_2_ [**(A)** here], the absolute minimum of |Δφ| is located on the upper left side of the heat map, where δ_1_ < δ_2_. When *d*_1_ < *d*_2_ **(B)** and *d*_1_ ≪ *d*_2_ **(C)**, the absolute minimum of |Δφ| lies on the lower right side, where δ_1_ > δ_2_. The maximum synchrony varies with both δ_*i*_ and *d*_*i*_, but it is never achieved when δ_1_ = δ_2_, regardless of the chosen coupling strengths. Other parameters are: α = −0.2; γ = −0.2; ω_0_ = 2; ω_1_ = 0; and λ_0_ = −0.5. The three-dimensional visualization of Figure 8 (surface plots) is illustrated in Supplementary Figures 1B–D.

**Figure 9:**
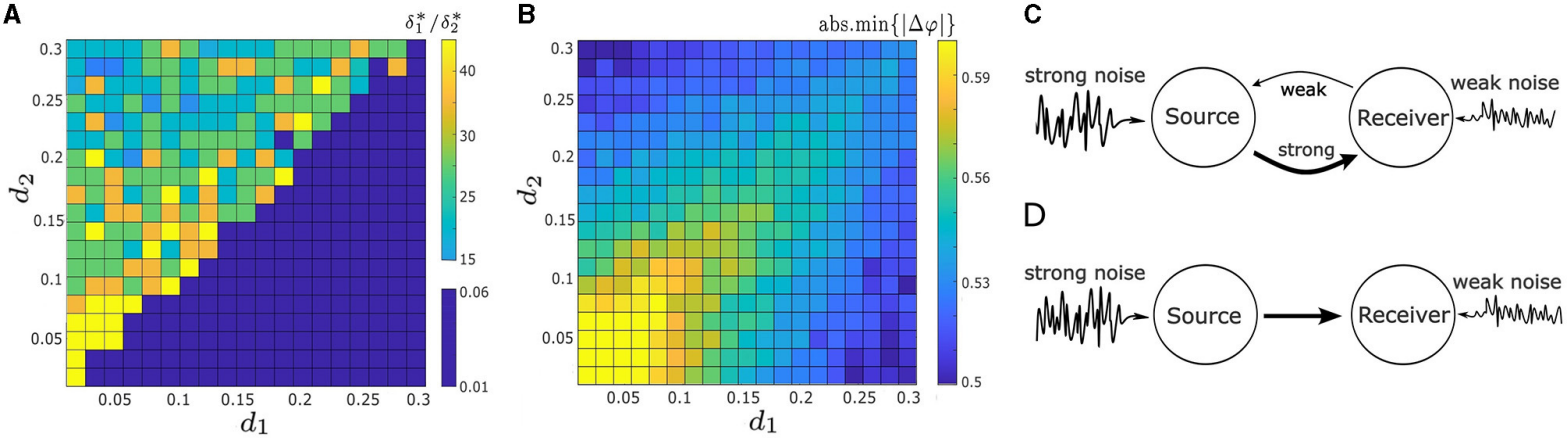
**(A)** Heat map illustrates the ratio of the optimal noise intensities, $\delta_{1}^{*}/\delta_{2}^{*}$, required to minimize |Δφ|, in relation to varying both coupling strengths, *d*_1_ and *d*_2_. **(B)** Heat map of the absolute minimum of |Δφ|, denoted as abs.min{|Δφ|}, as a function of *d*_1_ and *d*_2_. The maximal noise-induced synchrony is not achieveed when $\delta_{1}^{*}/\delta_{2}^{*}=1$ or when *d*_1_ = *d*_2_. **(C)** Schematic illustration of a two-neuron motif used to enhance noise-induced synchrony. One oscillator (receiver neuron) receives robust coupling from the other oscillator (source oscillator) along with minimal noise input. Conversely, the source neuron experiences minimal coupling from the receiver neuron but substantial noise input. **(D)** Two-neuron motif featuring a uni-directional connection, optimizing noise-induced synchrony. Other parameters in **(A, B)** are: α = −0.2; γ = −0.2; ω_0_ = 2; ω_1_ = 0; and λ_0_ = −0.5.

The bifurcation diagrams (i.e., Figures 1, 2) are generated using XPPAUT software (Ermentrout, 2012). All further analysis (i.e., Figures 3–**9**) is conducted using MATLAB. To simulate the SDEs in Equations 1, 2 we use the Euler-Maruyama method with time-step *dt* = 0.01 and arbitrary random initial conditions $x_{i}\left(0\right),y_{i}\left(0\right)\sim N\left(0,0.008^{2}\right)$, *i* = 1, 2. Due to the nature of white noise, noise-induced oscillations contain high-frequency fluctuations at very low or very high noise intensities, which poses difficulties in the computation of phase. Hence, to achieve more consistent numerical results a low-pass filter is applied to remove high-frequency fluctuations. The signal-to-noise ratio, β, and synchronization measures |Δφ|, *R*, and ρ in Equations 6–9, respectively, are averaged over *N* = 200 trials. The XPPAUT and MATLAB source code can be found at: https://github.com/TMUcode/CoupleNeurons.

## 3 Results

### 3.1 Bifurcation analysis

The dynamics of the deterministic system (δ_1_ = δ_2_ = 0) are examined in this section. We consider three cases and compare their bifurcation diagrams: single (i.e., uncoupled) oscillators with *d*_1_ = *d*_2_ = 0 (Figure 1A), two symmetrically coupled oscillators with *d*_1_ = *d*_2_ ≠ 0 (Figure 1B), and two asymmetrically coupled oscillators with *d*_1_ ≠ *d*_2_ (Figures 1C, D). When λ_0_ < 0, both oscillators in all three cases are quiescent, they rest at stable fixed points, as shown in Figure 1 (solid black line). Conversely, when λ_0_ > 0, the fixed points are destabilized (dashed black line), and stable periodic orbits emerge (solid blue line) in all three cases. Both isolated (Figure 1A) and coupled (Figures 1B–D) oscillators undergo a supercritical HB (denoted as HB_1_) at λ_0_ = 0. However, when the oscillators are coupled, the system exhibits a second HB (denoted as HB_2_) which leads to unstable periodic orbits (dashed blue line in Figures 1B–D).

When the oscillators are symmetrically coupled (e.g., *d*_1_, *d*_2_ = 0.05 in Figure 1B), the amplitudes of the periodic orbits generated by both oscillators are identical for each λ_0_ value, however, the unstable periodic orbits have an amplitude that is slightly less than the amplitude of the stable periodic orbits. When the oscillators are coupled asymmetrically (e.g., *d*_1_ = 0.1 and *d*_2_ = 0.01 in Figures 1C, D), the bifurcation diagram for one oscillator (Figure 1C) is identical to its counterpart in Figure 1B, whereas, the bifurcation diagram of the other oscillator (Figure 1D) shows a single difference: the amplitude of the unstable periodic orbit is near zero, which is much smaller counterpart in Figure 1B.

To further explore the effects of coupling on the deterministic system, we calculate the two-parameter bifurcation diagrams of our model by taking both the coupling strength, *d*_*i*_, and λ_0_ as control parameters. The trajectory of HB_1_ (solid blue line) in the two-parameter bifurcation diagram in Figure 2 indicates the transition of the system between the quiescent and oscillatory states in the deterministic regime remains the same (λ_0_ = 0) no matter the coupling strength. Moreover, the deterministic system is quiescent when λ_0_ < 0, but can be excited by an external stimulus (e.g., noise) to produce oscillations. Thus, we refer to the left side of HB_1_ as the excitable regime, and the right side of HB_1_ as the oscillatory regime.

Although HB_2_ is not a determinant factor of the excitability in the deterministic regime, we find that it has a relationship with the coupling strength: λ_0_ ≈ *d*_1_ + *d*_2_. Specifically, when the coupling is symmetric (*d*_1_ = *d*_2_), HB_2_ occurs at λ_0_ ≈ 2*d*_*i*_, *i* = 1or2, resulting in a line with a slope approximately equal to 1/2, as depicted by the dashed blue line in Figure 2A. For asymmetric couplings, such those obtained by varying *d*_1_ while fixing *d*_2_ = 0.05, HB_2_ appears as a straight line with a slope approximately equal to 1 (dashed blue line in Figure 2B), since λ_0_ ≈ *d*_1_ + 0.05. Another example of asymmetric coupling is obtained by varying *d*_2_ while fixing *d*_1_ = 0.05, which yields a straight line with the equation λ_0_ ≈ *d*_2_ + 0.05 (dashed blue line in Figure 2C).

### 3.2 Noise-induced oscillations

In this section, we study noise-induced oscillations. The control parameter λ_0_ is set to be in the excitable regime, for example, λ_0_ = −0.5. As a result, the deterministic system, with δ_1_ = δ_2_ = 0, displays damped oscillations that eventually converge to the fixed point (*x*_*i*_ = *y*_*i*_ = 0, *i* = 1, 2), as illustrated in Figure 3A. However, when the intrinsic noise, δ_*i*_*dη*_*i*_, is introduced, it can trigger repeated excursions from the stable fixed point which leads to oscillatory motion; otherwise known as noise-induced oscillations. Examples are presented in Figures 3B–D which display sample time series of *x*_1_ and *x*_2_ in the presence of the intrinsic noise stimulus with different intensities. When the noise intensity is small and symmetric (e.g. δ_1_ = δ_2_ = 0.01 in Figure 3B) there are intermittent periods of phase drift and phase locking; both in-phase and anti-phase. When the noise intensities become asymmetric by increasing one of the noise intensities, for example, δ_1_ = 0.01 and δ_2_ = 0.05 as in Figure 3C, the time series for *x*_1_ (blue line) and *x*_2_ (orange line) exhibit increased regularity and longer epochs of in-phase locking. Furthermore, if δ_2_ is increased further, for example, δ_2_ = 3 as in Figure 3D, the oscillations of *x*_2_ become less regular and more chaotic. Overall, these examples demonstrate that synchrony can be optimized by tuning just one of the noise intensities to be an intermediate value.

To determine an appropriate range of noise intensities, δ_1_ and δ_2_, we make use of the signal-to-noise ratio measure (Gang et al., 1993; Pikovsky and Kurths, 1997).


(6) $$\begin{array}{l} \beta =h_{p}{\left(\Delta \omega /\omega_{p}\right)}^{-1} \end{array}$$

where *h*_*p*_ and ω_*p*_ denote the height and central frequency of the power spectrum density peak of *x*, respectively, and Δω denotes the width of the power spectrum density peak at half-maximal power, $e^{-1/2}h_{p}$. We have computed the relationship between β and δ for a single λ−ω oscillator, (*x, y*), and present the results in Figure 4. When δ < 0.01, we find β is significantly small, indicating that the noise level is too weak. However, as the level of noise gradually increases, the β curve displays a peak around δ = 0.9, indicating that this noise intensity is optimal. For larger values of δ, specifically, when δ > 0.9, we observe a sharp decrease in β, suggesting that the noise intensity is now overpowering the regularity of oscillations. Therefore, based on these observations, we select the range 0.01 ≤ δ_1_, δ_2_ ≤ 5 for our analysis.

### 3.3 Noise-induced synchronization and asymmetric noise intensity

The results presented in Section 3.2 suggest that the synchrony of our system with asymmetric noise and uneven coupling can be optimized by adjusting the noise intensities, δ_1_ and δ_2_. To quantify the effects of noise on the synchrony of our model more rigorously, we introduce three measures of synchrony. The first measure is the absolute phase difference, denoted |Δφ|, which is defined as


(7) $$\begin{array}{l} |\Delta \varphi |=\frac{1}{T-t_{0}}\int_{t_{0}}^{T}|\varphi_{1}\left(t\right)-\varphi_{2}\left(t\right)|\;dt \end{array}$$

where φ_*i*_(*t*) is the phase of the *i*th oscillator. Since our model considers excitatory coupling, we focus on the in-phase dynamics of the oscillators, such that smaller values of |Δφ| correspond to a greater degree of synchrony. The second measure we use is the mean phase coherence, denoted *R*, which is defined as (Mormann et al., 2000; Rosenblum et al., 2001),


(8) $$\begin{array}{l} R=\sqrt{{\left(\frac{1}{T-t_{0}}\int_{t_{0}}^{T}\sin\Delta \varphi \;dt\right)}^{2}+{\left(\frac{1}{T-t_{0}}\int_{t_{0}}^{T}\cos\Delta \varphi \;dt\right)}^{2}} \end{array}$$

where Δφ = φ_1_ − φ_2_. The value of *R* ranges from 0 to 1, and larger values of *R* indicate a greater degree of synchrony. The third synchronization measure we use is the normalized synchronization index, denoted ρ, which is defined as (Rosenblum et al., 2001),


(9) $$\begin{array}{l} \rho =\frac{S_{max}-S}{S_{max}} \end{array}$$

where $S=-\overset{M}{\underset{k=1}{\sum }}p_{k}\ln p_{k}$ is the Shannon entropy, *S*_*max*_ = ln*M* is the maximum entropy, *p*_*k*_ refers to the probability of finding Δφ in the *k*th bin of the histogram, and *M* is the total number of bins. The quantity ρ is normalized to 0 ≤ ρ ≤ 1, and smaller values of ρ indicate a narrower distribution of Δφ and thus a greater degree of synchrony.

To begin we consider the effect of a single noise intensity on the degree of synchrony between two unevenly coupled oscillators. To achieve this, we set *d*_1_ ≠ *d*_2_, fix one noise intensity to be δ_1_ = 0.05, and vary the other noise intensity, δ_2_, from 0.01 to 5 and compute the level of synchrony using the three measures listed above. We present the results in Figure 5A, where the blue, orange, and black curves correspond to |Δφ|, *R*, and ρ, respectively. For weak δ_2_, both ρ and |Δφ| show a rapid decrease while *R* displays a rapid increase. These consistent results suggest that the level of synchrony is increasing. However, for strong δ_2_, we notice an opposite trend, where ρ and |Δφ| increase while *R* decreases, indicating a decrease in the level of synchrony.

Furthermore, synchrony is maximized at an intermediate noise intensity, which we find to be δ_2_ = 0.95 for all three measures in Figure 5A. Notice that it is close to the optimal noise intensity of a single oscillator as shown in Figure 4. These changes in synchrony may also be observed from the probability density functions of Δφ shown in Figure 5B. The dotted blue curve (labeled a), solid blue curve (labeled b), and dashed blue curve (labeled c) correspond to noise intensities δ_2_ = 0.05, 0.95, and3, respectively (i.e., points a, b, and c in Figure 5A). As δ_2_ increases from weak to intermediate levels, the center of the distribution of Δφ shifts from small negative values toward the point Δφ = 0, which represents in-phase synchronization. Moreover, the distribution of Δφ is centered at 0.08 at the optimal noise intensity δ_2_ = 0.95 in Figure 5A. Additionally, the width and peak of the distribution become narrower and higher, respectively, indicating a more concentrated distribution of phase differences between the two oscillators (i.e., a higher level of synchrony). As the noise intensity continues to increase, the center of the Δφ distribution remains almost unchanged, but its width and peak increase and decrease, respectively, which is indicative of a lower degree of synchrony.

Next, we investigate how both noise intensities, δ_1_ and δ_2_, affect the level of synchrony between two unevenly coupled oscillators. To systematically examine their influence, we generate heat maps, which represent the level of synchrony using the measures |Δφ| and *R* over the parameter space 0.01 ≤ δ_1_, δ_2_ ≤ 5, as illustrated in Figures 6A, B, respectively. In Figure 6A, warmer colors indicate larger values of |Δφ| (i.e., lower levels of synchrony), while cooler colors correspond to smaller values of |Δφ| (i.e., higher levels of synchrony). Note, a three-dimensional representation of Figure 6A is also presented in Supplementary Figure 1A. The heat map of *R* in Figure 6B corroborates the results obtained from Figure 6A, but with a different color scheme; warmer colors indicate higher levels of synchrony (i.e., larger *R* values), while cooler colors denote lower levels of synchrony (i.e., smaller *R* values). As such, we will primarily focus on Figure 6A. The highest level of synchrony (i.e., the absolute minimum of |Δφ|) is observed in the dark blue triangular region [where δ_1_ ∈ [0.01, 0.06], δ_2_ ∈ [0.1, 1], and δ_1_/δ_2_ ≈ 0.2]. A local minimum of |Δφ| is also observed in the lower right part of the heat map [where δ_1_ ∈ [0.8, 3] and δ_2_ ∈ [0.01, 0.015]], with a relatively high level of synchrony. Due to uneven coupling strengths (*d*_1_ = 0.3 and *d*_2_ = 0.01) used in Figure 6, the locations of the absolute and local minima are not symmetrical. Our numerical results further indicate a relationship between the coupling strength and the optimal noise intensity required to achieve the maximum level of synchrony (i.e., the absolute maximum of |Δφ|). For instance, as shown in Figure 6, oscillator 1 has a larger coupling strength than oscillator 2 (i.e., *d*_1_ > *d*_2_), thus requiring δ_1_ < δ_2_ to achieve the largest level of synchrony. In Section 3.5 we study the interplay of coupling and noise intensity and their effects on the synchrony in greater detail.

### 3.4 The effects of λ_0_ on synchronization

As explained in Section 2.1, our system operates in the excitable regime, λ_0_ < 0, where HB_1_ occurs at λ_0_ = 0. Previous research (e.g., Yu et al., 2009, 2021) has demonstrated that in excitable networks, the distance of the control parameter from a critical point (or excitation threshold) is negatively correlated with synchronization. Moreover, in this section, we investigate the effects (if any) of the control parameter λ_0_ on the level of synchrony. We only use |Δφ| as a measure of synchrony since it shows results consistent with ρ and *R*, as demonstrated in Figures 5, 6.

We begin by evaluating |Δφ| for various λ_0_ values within the excitable regime, λ_0_ < 0, as illustrated in Figure 7 (|Δφ| vs. δ_2_ with fixed δ_1_ = 0.1). The solid blue, dashed orange, and dotted black lines correspond to λ_0_ = −0.03, −0.5, and −1, respectively. As observed for the blue curve in Figure 5A, all three |Δφ| curves in Figure 7A exhibit a decreasing trend until they reach a minimum value, after which they start increasing again. This suggests that the level of synchrony can be optimized by adjusting the intrinsic noise intensity δ_2_. Furthermore, for a fixed δ_2_ within the weak intensity range (e.g., δ_2_ < 0.5 in Figure 7A), |Δφ| is smaller when λ_0_ is closer to zero. This indicates that in the weak noise regime synchronization is enhanced as λ_0_ approaches the excitation threshold λ_0_ = 0 (i.e., HB_1_ in Figures 1, 2). Conversely, when δ_2_ is fixed within the strong intensity range (e.g., δ_2_ > 1.5 in Figure 7A), |Δφ| increases as λ_0_ approaches the excitation threshold, resulting in lower levels of synchrony.

Furthermore, we observe that the minimum values of |Δφ| vary for different λ_0_, and the corresponding optimal noise intensities differ as well. The minimum |Δφ| values, denoted by min{|Δφ|}, are marked by black dots in Figure 7A. When λ_0_ is closer to the excitation threshold, our model achieves a greater degree of synchrony, and a smaller optimal δ_2_ is required to produce it (for fixed δ_1_ = 0.1). To explore this further, we plot the values of min{|Δφ|} and the sum of the optimal noise intensities, denoted $\delta_{1}^{*}+\delta_{2}^{*}$, for a range of λ_0_ values in Figures 7B, C, respectively. For λ_0_ values that are relatively far from the excitation threshold (e.g., λ_0_ < −0.3), the curve in Figure 7B is relatively flat and the curve in Figure 7C has a relatively low slope (≈−0.332). This indicates that min{|Δφ|} and the corresponding optimal intensities remain stable over this region of λ_0_. Hence, it follows that small changes in λ_0_ do not significantly affect the synchronization of our model when λ_0_ is far from the threshold λ_0_ = 0. However, when λ_0_ approaches the excitation threshold (e.g., −0.5 < λ_0_ < 0 in Figures 7B, C), min{|Δφ|} decreases exponentially, and $\delta_{1}^{*}+\delta_{2}^{*}$ decreases with a steeper slope (≈ −1.84) as $\lambda_{0}\to 0^{-}$. These findings suggest that by shifting λ_0_ closer to the excitation threshold, the noise-induced synchrony of our system can be enhanced, with smaller intensities of noise required to produce this effect.

### 3.5 The dual effects of uneven coupling and asymmetric noise on synchrony

In Section 3.3, we briefly described the relationship between the necessary coupling strength and the optimal noise intensity for attaining the maximum level of synchrony. Figure 6 illustrates that when the coupling strength *d*_1_ of oscillator 1 is greater than that of oscillator 2 (*d*_2_), achieving the greatest level of synchrony requires δ_1_ < δ_2_. In this section, we investigate how uneven coupling and asymmetric noise intensities interact with one another to influence synchrony. For the sake of simplicity, we set λ_0_ = −0.5 and use |Δφ| to quantify synchrony.

We begin by examining the change in |Δφ| as a function of δ_1_ and δ_2_ in four disparate coupling cases: *d*_1_ ≫ *d*_2_ (e.g., *d*_1_ = 0.3 and *d*_2_ = 0.01 in Figure 6A); *d*_1_ > *d*_2_ (e.g., *d*_1_ = 0.2 and *d*_2_ = 0.1 in Figure 8A); *d*_1_ < *d*_2_ (e.g., *d*_1_ = 0.1 and *d*_2_ = 0.2 in Figure 8B), and *d*_1_ ≪ *d*_2_ (e.g., *d*_1_ = 0.01 and *d*_2_ = 0.3 in Figure 8C). The four cases are also visualized in three dimensions using surface plots and presented in Supplementary Figure 1. Both heat maps and surface plots show that |Δφ| has both a unique absolute minimum and unique local minimum which correspond to points of increased synchrony. Furthermore, each panel in Figure 8 exhibits a relatively low degree of synchrony along the line δ_1_ = δ_2_ (i.e., a relatively larger |Δφ|), which is in agreement with our results in Figure 6. These observations suggest that noise-induced synchrony tends to be maximized by asymmetric noise intensities (i.e., δ_1_/δ_2_ ≠ 1) as opposed to symmetric noise intensities (i.e., δ_1_/δ_2_ = 1).

Figures 6A, 8 further indicate that the ratio of coupling strengths, *d*_1_/*d*_2_, determines whether the minimum point of |Δφ| is local or absolute. Let abs.min{|Δφ|} denote the absolute minimum of {|Δφ|} and $\delta_{1}^{*}/\delta_{2}^{*}$ denote the corresponding ratio of optimal noise intensities. We find that when *d*_1_ ≫ *d*_2_ or *d*_1_ > *d*_2_, abs.min{|Δφ|} is located on the upper left (i.e., δ_2_ > δ_1_) of the heat maps in Figures 6A, 8A, with $\delta_{1}^{*}/\delta_{2}^{*}\ll 1$. Conversely, when *d*_1_ < *d*_2_ or *d*_1_ ≪ *d*_2_, abs.min{|Δφ|} is located on the lower right (i.e., δ_1_ > δ_2_) of the heat maps in Figures 8B, C, with $\delta_{1}^{*}/\delta_{2}^{*}\gg 1$. Supplementary Figure 1 provides a clear depiction of the gradual transition of abs.min{|Δφ|} from the left side (with δ_1_ < δ_2_) to the right side (with δ_1_ > δ_2_) as *d*_1_/*d*_2_ changes from larger to smaller values.

To systematically explore the interaction and effects of the noise intensities and coupling strengths on synchrony, we consider the parameter space *d*_1_, *d*_2_ ∈ [0.01, 0.3] and δ_1_, δ_2_ ∈ [0.01, 0.3], and compute abs.min{|Δφ|} and $\delta_{1}^{*}/\delta_{2}^{*}$. We present the results in Figures 9A, B in the form of heat maps. The upper-left “triangle” of Figures 9A, B, where *d*_1_ < *d*_2_, corresponds to the absolute minimum points on the lower right of the heat maps in Figures 8B, C. And the lower-right “triangle” of Figures 9A, B, with *d*_1_ > *d*_2_, correspond to the absolute minimum points on the upper left of the heat maps in Figures 6A, 8A.

Figure 9A illustrates how the choice of coupling affects $\delta_{1}^{*}/\delta_{2}^{*}$. Previously, we have shown that the optimal $\delta_{1}^{*}/\delta_{2}^{*}$ does not equal 1 for some example pairs of *d*_1_ and *d*_2_ in Figures 6, 8. Here our result is more robust: noise-induced synchrony is never maximized at δ_1_/δ_2_ = 1 no matter the choice of coupling strengths *d*_1_ and *d*_2_. In particular, even when *d*_1_ = *d*_2_, $\delta_{1}^{*}/\delta_{2}^{*}\neq 1$. Figure 9A also indicates that there is a strong relationship between *d*_1_/*d*_2_ and $\delta_{1}^{*}/\delta_{2}^{*}$. Specifically, when *d*_1_/*d*_2_ < 1 (upper-left “triangle”), $\delta_{1}^{*}/\delta_{2}^{*}\in \left[15,35\right]\gg 1$; whereas, when *d*_1_/*d*_1_ > 1 (lower-right “triangle”), $\delta_{1}^{*}/\delta_{2}^{*}\in \left[0.025,0.04\right]\ll 1$. We also compute $\delta_{1}^{*}$ and $\delta_{2}^{*}$ for both *d*_1_/*d*_2_ cases. When *d*_1_/*d*_2_ < 1, the mean optimal noise intensities are $\bar{\delta_{1}^{*}}=0.3462$ and $\bar{\delta_{2}^{*}}=0.015$, which results in an average ratio of 0.3462/0.015 ≈ 23.08. And conversely, when *d*_1_/*d*_2_ > 1, $\bar{\delta_{1}^{*}}=0.015$ and $\bar{\delta_{2}^{*}}=0.3462$, which results in an average ratio of 0.015/0.3462 ≈ 0.043 (note that the latter ratio is the reciprocal the former).

Next, we examine how the coupling strengths influence the degree of synchrony of our system, as measured by abs.min{|Δφ|}. As in Figure 9B, abs.min{|Δφ|} exhibits symmetry with respect to the diagonal line *d*_1_ = *d*_2_. Along this line, abs.min{|Δφ|} decreases as both *d*_1_ and *d*_2_ increase. However, the smallest value of abs.min{|Δφ|} is not located along this line. This suggests that when the oscillators are symmetrically coupled, the degree of synchrony is positively correlated with the coupling strength *d*_1_ = *d*_2_, but does not lead to maximal synchronization.

In contrast, when the coupling strengths are asymmetric, abs.min{|Δφ|} decreases as the absolute difference between two coupling strength, |*d*_1_ − *d*_2_|, becomes larger, as seen in Figure 9B. Furthermore, the lowest values of abs.min{|Δφ|} occur at the upper-left and lower-right corners of Figure 9B, where one coupling strength is nearly zero and the other is the largest in the considered range. This indicates that a one-way coupling maximizes noise-induced synchronization between two coupled oscillators. Overall, synchrony is maximized when *d*_1_ ≪ *d*_2_ and δ_1_/δ_2_ ≈ 15 (the upper-left corner in Figures 9A, B), or *d*_1_ ≫ *d*_2_ and δ_1_/δ_2_ ≈ 1/15 (lower-right corner in Figures 9A, B).

Our findings indicate that the noise-induced synchrony between two coupled oscillators is enhanced in a specific scenario, as illustrated in Figure 9C. Specifically, one oscillator (referred to as the source neuron) experiences strong intrinsic noise and receives weak coupling input from the other oscillator (the receiver neuron). Conversely, the receiver neuron experiences weak intrinsic noise but receives substantial coupling input from the source neuron. The optimum noise-induced synchrony occurs in the extreme case, i.e., a uni-directional connection where the input from the receiver neuron to the source neuron is absent, as demonstrated in Figure 9D.

## 4 Discussion

In many investigations exploring the noise-inuced synchronization of coupled excitable neurons, assumptions of homogeneity or near-homogeneity (e.g., common noise, symmetric noise, and equal coupling strength) are undertaken. To move beyond these seemingly unrealistic setups, our study considers a heterogeneous configuration through the introduction of independent noise sources with varying intensities and distinct coupling strengths for each neuron. We investigate the synchronous dynamics of the most representative neuron motif, characterized mathematically as a pair of coupled λ−ω oscillators in the heterogeneous context. These neural oscillators remain are quiescent in the absence of noise but may be excited by adding an intrinsic noise stimulus. Our results show that noise can induce synchronization in coupled oscillators, and this synchronization is enhanced by positioning the model closer to the bifurcation point (i.e., excitation threshold), which is consistent with previous studies (e.g., Yu et al., 2006, 2008, 2021; Thompson et al., 2012). In previous research, the noise-induced synchrony of excitable systems has been studied extensively with a focus on symmetrical interactions between oscillators (e.g., Neiman et al., 1999; Rosenblum et al., 2001; Freund et al., 2003). However, in biological systems, interactions between oscillators are often asymmetric, and thus, the assumption of symmetric interactions may be overly restrictive (Cimponeriu et al., 2003; Sheeba et al., 2009).

We investigate the impacts of two sources of heterogeneity (uneven coupling and asymmetric intrinsic noise) on the noise-induced synchrony of two coupled oscillators. Our results indicate that when the two noise intensities, δ_1_ and δ_2_, are asymmetric (i.e., δ_1_/δ_2_ ≠ 1), synchrony is promoted. We further find that the synchronization between two coupled oscillators is optimized when the absolute difference between the coupling strengths, |*d*_1_ − *d*_2_|, is as large as possible, irrespective of the coupling strengths selected. Furthermore, we have identified a correlation between the asymmetry of coupling and the intensity of noise required to maximize it. Specifically, when *d*_1_/*d*_2_ < 1, it leads to $\delta_{1}^{*}/\delta_{2}^{*}>1$, whereas, when *d*_1_/*d*_2_ > 1, it leads to $\delta_{1}^{*}/\delta_{2}^{*}<1$.

Our results suggest that there is a strong relationship between uneven coupling and asymmetric noise in coupled oscillators, which have potential applications in various real-world problems that exhibit asymmetry in the interactions between oscillators, such as cardio-respiratory electroencephalogram (EEG) interactions (Paluš and Stefanovska, 2003; Sheeba et al., 2009), optical communication systems, the detection of radar signals in presence of channel noise (Tsang and Lindsey, 1986), and neuronal dynamics (Singer, 1999; Sheeba et al., 2009). Moreover, our results indicate that two-neuron motifs with uni-direction (i.e., one-way) connections may exhibit a greater propensity for in-phase synchronization than two-neuron motifs with bi-directional (i.e., two-way) connections, which reinforces our understanding of the functional significance of network motifs in neuronal dynamics.

However, further investigations are necessary to explore the relationship between the ratios *d*_1_/*d*_2_ and δ_1_/δ_2_ more extensively. An extension of our current work may include investigating: (1) the role of asymmetric noise and coupling in the anti-phase synchronization of coupled oscillators by considering inhibitory coupling (i.e., *d*_*i*_ < 0 for *i* = 1or2); and (2) investigation of the role of noise and coupling asymmetries in other common network motifs, such as three-neuron feed-forward-loops.

The HB is one of the most common bifurcation schemes as it describes the appearance or disappearance of regular orbits with a slight change of a parameter. In this study, we employ the normal form of HB to investigate a specific scenario: noise-induced synchrony near a supercritical Hopf point. By focusing on this particular region, we aim to uncover the underlying mechanisms that lead to synchronized oscillations in physical or biological systems. Our results not only shed light on the phenomena of interest but also offer potential explanations or predictions for various systems that undergo similar shifts from a quiescent state to sustained oscillations. However, it is important to acknowledge that different systems may exhibit other bifurcation structures, necessitating further investigation to comprehensively understand their dynamic behavior.

## Data availability statement

The original contributions presented in the study are included in the article/Supplementary material, further inquiries can be directed to the corresponding author.

## Author contributions

GJ: Formal analysis, Investigation, Methodology, Software, Visualization, Writing - original draft, Writing - review & editing. NY: Conceptualization, Formal analysis, Funding acquisition, Investigation, Methodology, Supervision, Visualization, Writing - original draft, Writing - review & editing.
