# Supplementary material for: Noise-induced synchrony of two-neuron motifs with asymmetric noise and uneven coupling
Source: Front Comput Neurosci. 2024 Feb 23;18:1347748. doi: 10.3389/fncom.2024.1347748 (PMC10920254; doi:10.3389/fncom.2024.1347748)
Supplement: Supplementary file 1 [file Data_Sheet_1.pdf]

Supplementary Material

Noise-induced synchrony of two-neuron motifs with asymmetric noise and uneven coupling

Gurpreet Jagdev, Na Yu*

*** Correspondence:** Corresponding Author: nayu@torontomu.ca

Supplementary Figure

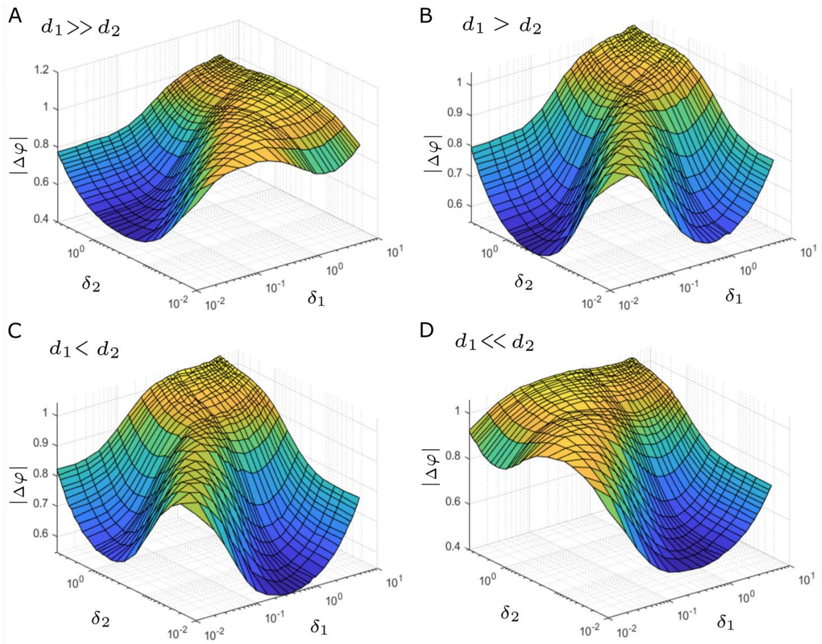

Supplementary Figure 1. Surface plots of $|\Delta\varphi|$, as a function of δ_1 and δ_2 for four cases of coupling strengths: **(A)** $d_1 \gg d_2$ (with d_1=0.3, d_2=0.01); **(B)** $d_1 > d_2$ (with d_1=0.2, d_2=0.1); **(C)** $d_1 < d_2$ (with d_1=0.1, d_2=0.2); **(D)** $d_1 \ll d_2$ (with d_1=0.01, d_2=0.3). When $d_1 \gg d_2$ (panel A), the absolute minimum of $|\Delta\varphi|$ is situated on the left side of the surface plot (with $\delta_1 < \delta_2$). As d_2 increases but d_1 decrease (from panel B to panel C then to panel D), the absolute minimum of $|\Delta\varphi|$ gradually shifts to the right side of the surface plot (with $\delta_1 > \delta_2$). Other parameters are: α=-0.2; γ=-0.2; ω_0 =2; ω_1=0; and λ=-0.5. This figure provides the three-dimensional visualization of the heat maps presented in Fig. 6A and Fig. 8.
